# A suspicion index tool to aid the diagnosis and treatment of ASMD

**DOI:** 10.1186/s13023-026-04328-z

**Published:** 2026-03-27

**Authors:** Anna-Maria Wiesinger, Georg Zimmermann, Wanda Lauth, Nicole Muschol, Dorothea Möslinger, Vassiliki Konstantopoulou, Sabine Scholl-Bürgi, Daniela Karall, Roberto Giugliani, Eugen Mengel, Elmar Aigner, Daniel Weghuber, Florian B. Lagler

**Affiliations:** 1 Institute of Inherited Metabolic Diseases, Paracelsus Medical University, Strubergasse 21, Salzburg, 5020 Austria; 2 Biostatistics and Big Medical Data, IDA Lab, Paracelsus Medical University, Salzburg, Austria; 3 International Center for Lysosomal Disorders (ICLD), Department of Pediatrics, University Medical Center Hamburg-Eppendorf, Hamburg, Germany; 4 Department for Pediatrics and Adolescent Medicine, Inborn Errors of Metabolism, Medical University of Vienna, Vienna, Austria; 5 Department of Pediatrics, Medical University of Innsbruck, Innsbruck, Austria; 6 Department of Genetics, UFRGS, Medical Genetics Service and Biodiscovery Laboratory, HCPA, DASA Casa dos Raros, Porto Alegre, Brazil; 7 SphinCS GmbH, Institute of Clinical Science for LSD, Hochheim, Germany; 8 European Reference Network for Hereditary Metabolic Diseases, MetabERN, Udine, Italy; 9 Obesity Research Unit, Paracelsus Medical University, University Hospital Salzburg, Salzburg, Austria; 10 Department of Pediatrics, Paracelsus Medical University, University Hospital Salzburg, Salzburg, Austria; 11 Department of Artificial Intelligence and Human Interfaces, Faculty of Digital and Analytical Sciences, Paris Lodron University, Salzburg, Austria

**Keywords:** Acid sphingomyelinase deficiency, Diagnosis, Suspicion index, Risk prediction score, Hepatosplenomegaly

## Abstract

**Background:**

The diagnosis of acid sphingomyelinase deficiency (ASMD, Niemann Pick Type A, A/B, B) is frequently delayed by years, because of its heterogeneous and often unspecific clinical features. The involvement of multiple organs including the musculoskeletal and central nervous systems, poses a challenge for accurate diagnosis. To address this, we developed a suspicion index tool (SIT) for healthcare professionals to enable early and accurate diagnosis of ASMD.

**Methods:**

Our methodological approach encompasses five key steps: (i) literature research on ASMD symptomatology, (ii) retrospective expert chart review of international ASMD centers, (iii) retrospective statistical analysis, (iv) development of an individual risk prediction score via random forest regression, and multinomial modeling, (v) internal validation of the tool via bootstrap resampling.

**Results:**

Data were collected from 908 patients (48 cases, 52 controls, and 808 non-cases) from eight expert centers. Visceral symptoms emerged as strong indicators of ASMD, particularly isolated unexplained splenomegaly (100% of cases vs. 71% of controls and 0.4% of non-cases) and hepatomegaly (92% of cases vs. 56% of controls and 0.4% of non-cases). Respiratory symptoms, thrombocytopenia, and hypercholesterolemia were also identified as significant indicators. These variables were selected for inclusion in the final SIT using a best subset selection algorithm. Each variable composition was evaluated via extensive repetitions. Additionally, expert input was sought to assess the significance of selected variables. The SIT demonstrated superior accuracy, sensitivity, specificity, and internal validity, confirming its reliability.

**Conclusions:**

The SIT is currently under development as a web-based platform for facilitating the diagnosis of ASMD and other treatable diseases in at-risk populations.

**Supplementary Information:**

The online version contains supplementary material available at 10.1186/s13023-026-04328-z.

## Take-home message

Early diagnosis of ASMD is crucial but often delayed. Our newly developed innovative diagnostic tool, which leverages a large patient dataset and advanced statistical and machine learning techniques, can improve the accuracy and reliability of ASMD diagnosis by considering multiple symptoms and enabling patient participation.

**Supplementary Information:**

The online version contains supplementary material available at 10.1186/s13023-026-04328-z.

## Introduction

Acid sphingomyelinase deficiency (ASMD), also known as Niemann Pick disease (NPD types A and B), is a rare, progressive autosomal recessive lysosomal storage disorder (LSD). Deficiency of the lysosomal enzyme acid sphingomyelinase (ASM) is caused by pathogenic variants in the sphingomyelin phosphodiesterase 1 (*SMPD1*) gene [1, 2]. Reduced or absent enzyme activity ultimately results in progressive cell and tissue damage, due to the accumulation of sphingomyelin and other lipids – especially in cells of the monocyte-macrophage system [3]. The level of residual ASM activity, which is determined by the type of pathogenic *SMPD1* variant, affects disease severity and functional impairment.

Consequently, the clinical phenotype of ASMD is highly heterogeneous, with neurovisceral (NPD-A and NPD A/B) or visceral (NPD-B) manifestations and unspecific clinical features. The involvement of different organs, the musculoskeletal system, and the central nervous system (CNS) represents a diagnostic challenge, especially in infantile neurovisceral ASMD (NPD-A) and chronic neurovisceral ASMD (NPD-A/B). Thus, the diagnosis of ASMD patients is frequently delayed by months to years, creating a diagnostic dilemma [4].

Early and accurate diagnosis is key for appropriate management and of utmost importance for patients with a chronically progressive disease where therapeutic options are available. In 2022, the first treatment for ASMD patients received a positive Committee for Medicinal Products for Human Use opinion in the European Union, the United States, and other countries. This enzyme replacement therapy (ERT) is indicated for the treatment of non-CNS manifestations of ASMD in adult and pediatric patients [5].

As the treatment of ASMD is no longer limited to symptomatic medication and supportive care, rapid action is required for the benefit of patients. Clinical trials highlight the need for an early diagnosis to achieve a better outcome [6]. Furthermore, Wijburg et al. and Pineda et al. demonstrated the clinical use of a suspicion index tool (SIT) for another lysosomal storage disorder, Niemann Pick disease type C (NPD-C), to support healthcare professionals, especially physicians with limited knowledge. This tool has ultimately led to improved detection rates for NPD-C, detection at an early age, and timely initiation of therapy [7, 8].

On the basis of validated tools for the NPD-C we developed a quantitative, evidence-based and innovative scoring system for ASMD. This paper reports the first SIT in ASMD, which is a low-threshold but effective measure for use in clinical practice.

## Methods

Our methodical approach consisted of five key steps: (i) literature research on ASMD symptomatology, (ii) retrospective expert chart review with international ASMD centers, (iii) retrospective and statistical analysis, (iv) development of an individual risk prediction score utilizing random forest regression and multinomial modeling, and (v) internal validation of the tool via bootstrap resampling.

### Literature analysis

A comprehensive literature review via Medline was conducted between October 2021 and December 2022 to collect data concerning the underlying clinical symptomatology. Published data, clinical studies, and case reports referring to confirmed ASMD cases were compiled. Therefore, the focus was on understanding which organ systems are affected, in what way, and to what extent. Furthermore, research has been conducted to determine which manifestations are more characteristic of each subtype of ASMD (NPD A, NPD A/B, NPD B).

### Expert chart review

A retrospective expert chart review was designed to gather clinical data from different international ASMD centers, between January 2022 and December 2022. Validated questionnaires were used [7] and adapted for ASMD, on the basis of a literature chart review. The questionnaire for the international expert chart review can be found in Suppl. Info. 1.

In addition to the collection of confirmed ASMD cases, non-cases, and control data were collated. This step was essential for the analysis of differential diagnostics. The study population was classified into three different patient groups:

1. ASMD cases: confirmed ASMD positive by molecular and clinical assessment with pathogenic variants in the sphingomyelin phosphodiesterase 1 gene (*SMPD1*).
  - NP-A cases: infantile neurovisceral ASMD cases associated with p.R498L, p.L304P and p.F333Sfs*52 variants.
  - NP-A/B cases: chronic neurovisceral ASMD associated with p.Q294K and p.W393G variants.
  - NP-B cases: chronic visceral ASMD associated with p.R610del, p.P323A, p.P330R and p.W393G variants.
2. ASMD non-cases: confirmed to be ASMD negative by molecular and clinical assessment.
3. Control cases: characteristic clinical symptoms of ASMD, but not suspected of having the disorder - patients with the confirmed lysosomal disease NPD-C, or Gaucher disease type I - were included in the final analysis.

For all cases, non-cases, and controls, one questionnaire was completed by a responsible and competent healthcare professional. All clinical cases were anonymized. As such, this study does not involve human subjects and therefore does not meet the criteria for research involving human participants, as defined by the Declaration of Helsinki. Consequently, no ethical approval was needed – however, an application has been submitted to the ethical committee in Salzburg (1126/2021).

### Data processing and statistical analysis

Data export and further processing were performed via the statistical software package R. A two-sided significance level of α = 0.05 was applied. The descriptive statistical analysis included several domains, such as family history, and sociodemographic, or individual signs and symptoms.

### Statistical analysis

To describe the characteristics of, and illustrate the symptomatology between groups, the minimum, first quartile, median, mean, standard deviation, third quartile, and maximum were calculated for metric values. Absolute and relative frequencies were calculated for binary, and nominal values, respectively. For the statistical analysis, Fisher exact test was applied to detect significant differences among individual symptoms in the three patient groups. Owing to the high number of related tests, the Bonferroni-Holm method was applied to correct for multiplicity. Box plots and bar plots were created for visualization.

### Development of a prediction model

We developed our ASMD SIT based on a multinomial model via a neural network [9]. This approach enables the prediction of ASMD cases, and distinguishes between control and non-cases. Owing to the high number of symptoms present, for the a priori variable selection, those demonstrating a highly significant difference (*p* < 0.001) via Fisher’s exact test were used for the development of our model.

In the next step, our dataset was divided into training (70%) and test (30%) sets. The multinomial model was constructed using the former with highly significant variables. The most important variables were subsequently identified via best subset selection. This process was repeated 1000 times, to avoid additional overfitting for different compositions of the training set. A key concept in the development of a prediction model is the number of events per predictor variable (EPV) [10]. A minimum of 10 EPVs has been advocated for the development of binary or time-to-event prediction models [11]. Thus, combinations with a maximum of four variables were considered in our model.

In addition, a random forest regression was set up via a similar approach as outlined above [12]. This method is more flexible in its modeling and can be used for practical comparisons with the abovementioned multinomial model. For the selection of variables, their importance was determined on the basis of the mean decrease in impurity (over 1000 repetitions), and the four most important variables were included in the final model. This model was subsequently compared with the best subset selection approach described above. The results were similar. Therefore, it was decided to focus on reporting the results of the multinomial model only, because obviously, the more flexible random forest model did not reveal any additional insights.

### Validation of the model

The performance of the SIT was evaluated by assessing accuracy, sensitivity, and specificity. These values were calculated iteratively by bootstrapping to obtain more robust values for different compositions of training and test sets, as well as facilitating the evaluation of precision through the calculation of confidence intervals.

## Results

### Patient characteristics

Eight international expert centers were included in the retrospective chart review. Data were collected from 908 patients: 48 cases, 52 controls, and 808 non-cases. The cases included 30 ASMD patients affected by type B, 16 patients with type A/B, and 2 patients with type A disease. The mean age at symptom onset varied widely among all three groups with 8.7 years of age (y) in the cases, 11.6 y in the controls, and 34.9 y among non-cases. The mean ages at data collection was 20.4 y, 22.9 y, and 14.0 y, respectively. Table 1 below describes the demographic data.

**Table 1:** Summary of patient demographics

	ASMD cases (*n* = 48)	Controls (*n* = 52)	Non-cases (*n* = 808)	All patients (*n* = 908)
Gender, *n* (%)				
Male	26 (54%)	24 (46%)	520 (64%)	570 (63%)
Female	22 (46%)	28 (54%)	288 (36%)	338 (37%)
Age at symptom onset, y mean, (SD)	8.6 (12.7)	11.6 (10.9)	34.9 (19.3)	18.4 (14)
Current age, y mean, (SD)	20.4 (15.9)	22.9 (14.8)	14.0 (5.4)	19.1 (5.4)
ASMD family history	10 (21%)	0	0	10 (1%)
Parent or sibling with ASMD, n (%)	9 (19%)	0	0	9 (1%)
Cousin with ASMD, n (%)	1 (2%)	0	0	1 (0.1%)

### Key symptomatology

The key clinical signs and symptoms of ASMD were defined as those observed in more than 5% of the ASMD patients (see Table 2). Overall, visceral symptoms were the strongest indicators of ASMD, especially isolated unexplained splenomegaly and hepatomegaly.

**Table 2:** Summary of symptomatology observed in > 5% of ASMD cases; among neurological, visceral, and skeletal features

	Cases, *n* (%)	Controls, *n* (%)	Non-cases, *n* (%)	*P* value	Odds ratio
Neurological					
Learning disability	19 (40%)	13 (25%)	0	0.14	0.51
Muscular hypotonia	10 (21%)	15 (29%)	0	0.49	1.50
Peripheral neuropathy	10 (21%)	3 (6%)	1 (0.1%)	0.04	0.24
Loss of deep tendon reflexes	9 (19%)	5 (10%)	0	0.25	0.46
Macular halo	9 (19%)	0	0	≤ 0.001	0.01
Loss of skills	8 (17%)	17 (33%)	0	0.07	2.41
Ocular “cherry red spot”	8 (17%)	0	0	0.01	0.01
Ataxia	7 (15%)	26 (50%)	0	≤ 0.001	5.75
Psychiatric symptoms	7 (15%)	10 (19%)	0	0.60	1.39
Dysphagia	5 (10%)	26 (50%)	1 (0.1%)	≤ 0.001	8.41
Visceral					
Splenomegaly	48 (100%)	37 (71%)	3 (0.4%)	≤ 0.001	0.01
Hepatomegaly	44 (92%)	29 (56%)	3 (0.4%)	≤ 0.001	0.12
Interstitial lung disease	32 (67%)	3 (6%)	0	0.03	0.01
Thrombocytopenia (with bleeding tendencies)	27 (56%)	2 (4%)	4 (0.5%)	≤ 0.001	0.03
Mixed dyslipidemia with low HDL-C	24 (50%)	6 (12%)	806 (100%)	≤ 0.001	0.13
Recurrent respiratory tract infections	18 (38%)	0	0	≤ 0.001	0.01
Abnormal liver function test	15 (31%)	0	0	≤ 0.001	0.01
Diarrhea	15 (31%)	5 (10%)	0	0.01	0.24
Cholestatic jaundice	14 (29%)	2 (4%)	0	≤ 0.001	0.10
Portal hypertension	12 (25%)	0	0	≤ 0.001	0.01
Aspiration pneumonia	11 (23%)	1 (2%)	0	≤ 0.001	0.07
Liver fibrosis	11 (23%)	1 (2%)	1 (0.1%)	≤ 0.001	0.07
Feeding difficulties	6 (13%)	24 (46%)	0	≤ 0.001	5.89
Cardiac valve disease	3 (6%)	2 (4%)	0	0.67	0.60
Skeletal					
Growth retardation in childhood	24 (50%)	9 (17%)	0	≤ 0.001	0.21
Reduced bone density (with pathologic fractures)	13 (27%)	2 (4%)	0	≤ 0.001	0.11
Bone and joint pain	10 (21%)	3 (6%)	4 (0.5%)	0.04	0.24

In addition, strong indicators of ASMD were symptoms of the respiratory tract, particularly interstitial lung disease, and recurrent respiratory tract infections. A further commonly presented visceral symptom of ASMD was thrombocytopenia with bleeding tendencies. There was poor differentiation between cases, controls, and non-cases in terms of the occurrence of dyslipidemia, with low high-density lipoprotein (HDL) and high triglyceride (TG) and low-density lipoprotein (LDL) levels. Age might be a confounding factor for lipid levels, as lipid metabolism can vary significantly with age. In our study, the non-cases group was significantly older than the other two groups were, which could influence the observed lipid levels.

Overall, neurological and musculoskeletal symptoms were reported at a lower frequency than visceral symptoms were reported. Learning disabilities, followed by muscular hypotonia and peripheral neuropathy were the most prominent CNS signs in this ASMD patient dataset with > 20% each. Growth retardation was highly suggestive of ASMD with 50% vs. 17% in controls and 0% in non-cases. Another commonly presented skeletal sign of ASMD in our dataset was reduced bone mineral density with pathological fractures and bone and joint pain.

Several symptoms that occurred at a low frequency were highly specific to ASMD patients. In addition to recurrent respiratory infections, as outlined above, macular halos, cherry red perimacular retinal stigmata, portal hypertension, and abnormal liver function tests have been reported only in our cohort of ASMD patients.

A sign of high specificity for ASMD was the family relationship. Overall, 10 ASMD patients reported having an affected parent, sibling, or cousin. None of the ASMD non-cases or controls reported a first or second-degree family history.

### Development and validation of a risk prediction score

The variables were selected via best-subset selection using a multinomial model, where variables with a p-value of < 0.01 were considered. The combinations of the four variables with the best performance were included in the SIT were thrombocytopenia with bleeding tendencies, interstitial lung disease, mixed dyslipidemia with low HDL-C, hepatomegaly, and splenomegaly. We combined hepatomegaly and splenomegaly into a single variable due to their frequent co-occurrence in the majority of patients with ASMD, reflecting the disease’s systemic lysosomal storage pathology. This combination was also supported by clinical observation and data review, where isolated hepatomegaly without splenomegaly was exceedingly rare. Thus, merging these signs into one variable simplified the scoring system without compromising its diagnostic sensitivity or specificity.

**Fig. 1:**
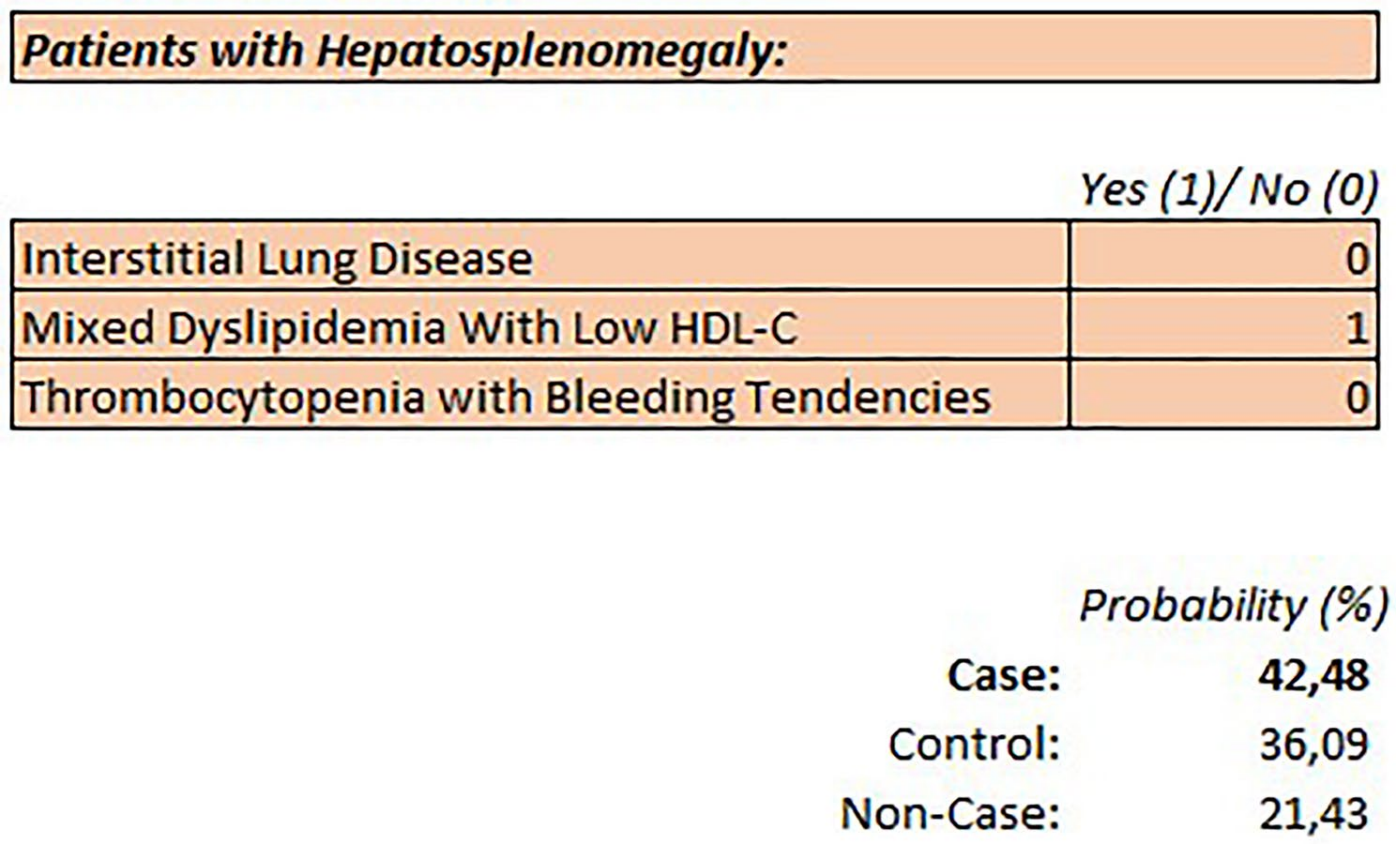
ASMD Suspicion Index Tool (SIT), which is based on a multinomial model via a neuronal network; four variables are included with hepatosplenomegaly as a prerequisite; the SIT provides a specific probability (%) for having ASMD which is based on the presence (1) or absence (0) of three symptoms; here an example is given in a patient with enlarged liver and spleen, and abnormal cholesterol levels, resulting in a probability of having ASMD of 42,48%

Figure 1 presents the proposed SIT for use by clinicians, and patients to calculate a total ASMD prediction score. It is expected to work through the selection of signs and symptoms by assigning the presence of a symptom with the numbers “1” and “0” for a nonoccurrence. The final risk prediction score is automatically calculated via an underlying formula. In addition, we used a straightforward approach and applied the variable hepatosplenomegaly as a prerequisite for using the tool. Thus, we combined a targeted diagnosis with a targeted audience.

**Fig. 2:**
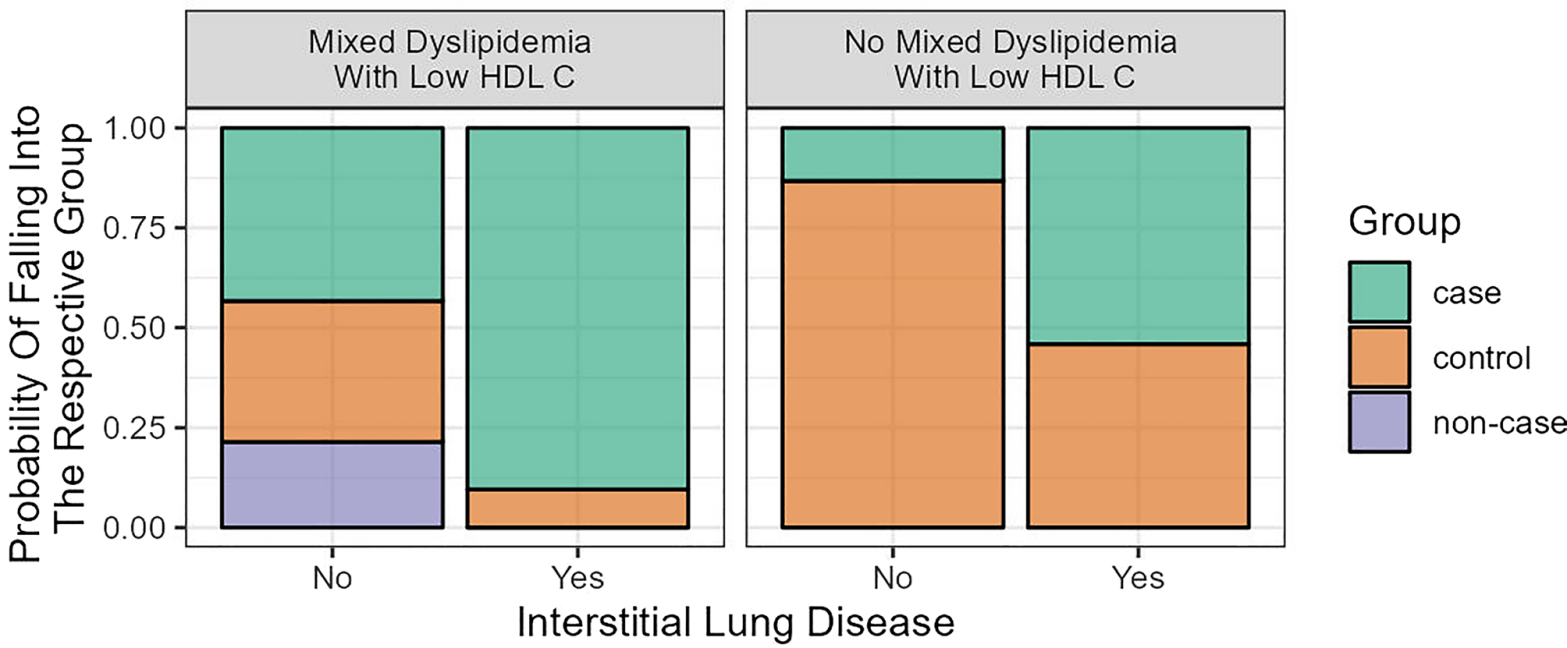
Probabilities for the combination of different variables; hepatosplenomegaly as a prerequisite with (non-) presence of interstitial lung diseases and (non-) presence of dyslipidemia; e.g.: the probability of having ASMD (% case) in a hepatosplenomegaly patient with interstitial lung disease and mixed dyslipidemia is approximately 90%, whereas having no interstitial lung disease results in approximately 45%

The simplicity of the diagnostic pathway is highlighted by this example:

The tool demonstrates a good ability to differentiate ASMD from other conditions with hepatosplenomegaly, which is a common sign of lysosomal storage disorders. Figure 2 underscores the effectiveness of the tool in identifying ASMD cases based on specific symptom clusters rather than relying solely on individual symptoms or a broad range of nonspecific symptoms.

The cutoff prediction score for a high risk of ASMD was assigned on the basis of sensitivity and specificity analyses of (i) cases vs. controls and (ii) cases vs. non-cases. A median total risk prediction score of 50% was calculated for ASMD cases.

**Fig. 3:**
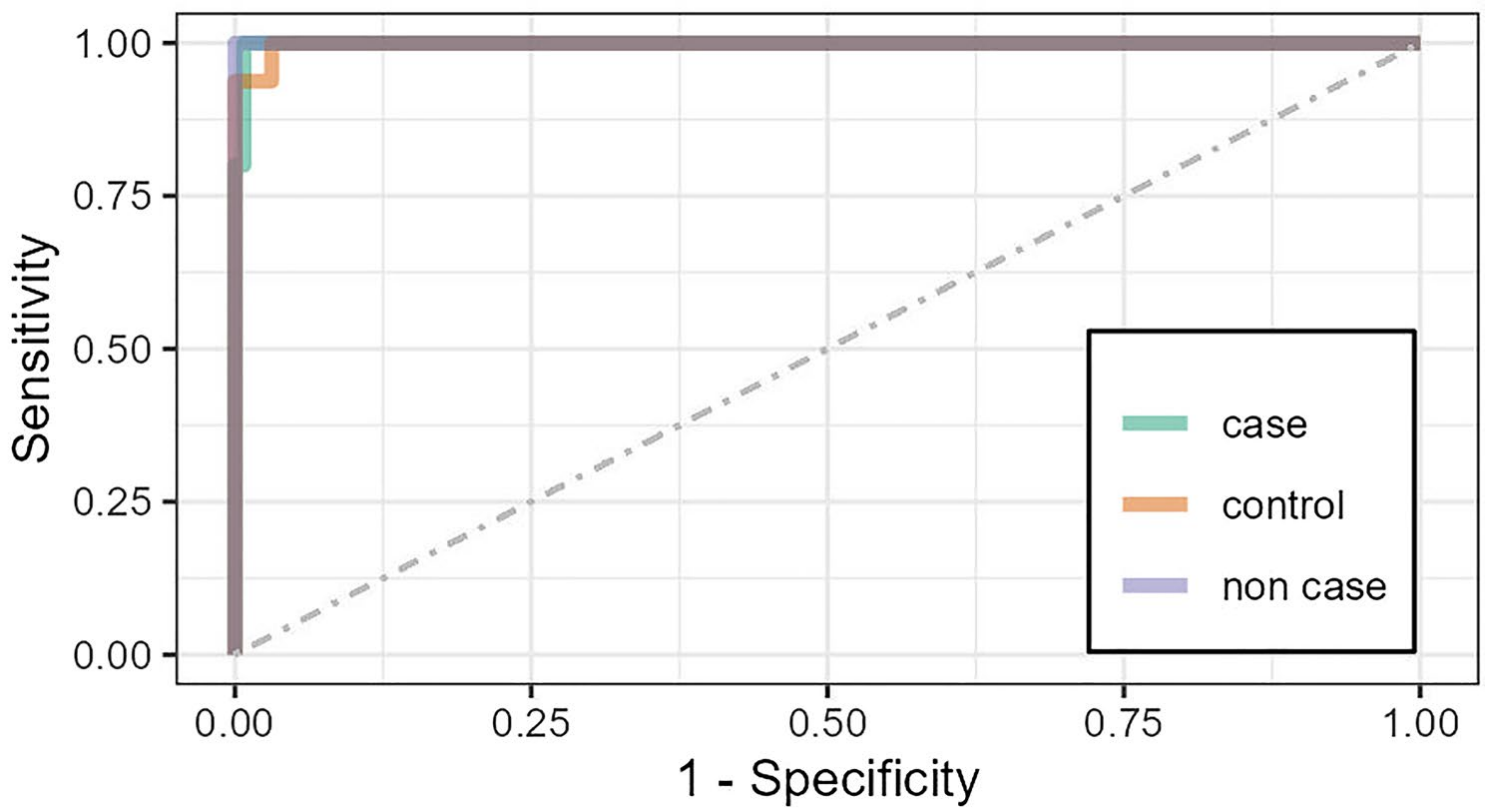
Receiver operating characteristic (ROC) analysis for risk prediction scores: cases vs. controls vs. non-cases with the combination of the four variables that occur in more than 70% of the best subset selection

A bootstrap approach and receiver operating characteristic (ROC) analysis were used for internal validation, which demonstrated very good accuracy, sensitivity, and specificity, confirming its reliability (Fig. 3).

## Discussion

The recent approval of ERT in 2023 for ASMD has revolutionized the treatment landscape for this devastating disease. Significant improvements in severe disease manifestations such as splenomegaly, hepatomegaly, or reduced lung diffusing capacity have been reported [13]. Crucially, initiating therapy early is paramount to prevent irreversible damage. However, most ASMD patients experience significantly delayed diagnoses, averaging approximately 5 years [4]. Despite longstanding recognition of the clinical picture of ASMD and numerous awareness and diagnostic initiatives with high-performing symptom-checker platforms, progress in achieving earlier and more reliable diagnoses has been limited [14–17].

In this study, we developed an innovative diagnostic tool using data from a comprehensive cohort of 908 patients, the largest collection of ASMD cases, controls, and non-cases to date. Our SIT (i) defines the target audience, (ii) suggests further diagnostic questions that are relevant not only for ASMD but also for other rare differential diagnoses, (iii) provides information when diagnostic evaluation for ASMD should be initiated, and (iv) allows patients to actively participate through self-completion of a questionnaire.

Delayed diagnosis results from various factors, including the lack of widespread newborn screening (NBS), rarity, heterogeneity, chronic progression, and overlapping clinical manifestations with other diseases. This contributes to late diagnosis, misdiagnosis, and reliance on few expert centers. Knowledge of which symptoms should prompt diagnostic evaluation and which professional groups to inform is crucial to shorten the diagnostic odyssey [3, 18].

Several studies have demonstrated that diagnostic support tools can facilitate accurate diagnoses on the basis of multiple symptoms with high reliability. This is especially critical in the context rare diseases. Prominent tools have been in existence for years [7, 8, 19–21]. Despite the recognized hurdles, projects aimed at improving the diagnostic process of ASMD have yet to be implemented.

We successfully developed a statistical tool to aid initial ASMD diagnosis. Our approach distinguishes this SIT by offering several advantages and special features. First, the use of a multinomial model allows for the simultaneous consideration of multiple symptoms and their combinations, enabling a more comprehensive assessment of the diagnostic landscape. This is particularly advantageous in the context of ASMD, where symptoms may vary widely among patients and across different disease types. Second, the incorporation of a neural network enhances the tool’s ability to identify complex patterns and relationships within the data. Neural networks excel at capturing nonlinear dependencies and interactions, which are common in medical datasets characterized by multifaceted clinical presentations [9, 22]. By leveraging advanced statistical techniques and machine learning algorithms, we have developed a novel tool that addresses an unmet need in the field of rare disease diagnostics. Additionally, our SIT benefits from a diverse dataset comprising heterogeneous patient information from America and Europe, enhancing its robustness and generalizability.

Our literature analysis identified 246 ASMD cases out of seven reports [23–29], providing a solid foundation for our study. The data collection via expert chart review, which included 48 ASMD cases, further underlines the value of our sample, which, to our knowledge, is the most comprehensive report of all three ASMD types. Our findings align with those of previous studies in which visceral manifestations were identified as the most common and discriminative symptoms across all ASMD types. While splenomegaly and hepatomegaly were recognized with a similarly high prevalence (≥ 90%) via literature analysis and expert chart review, both manifestations of interstitial lung disease (58% vs. 67%) and thrombocytopenia (41% vs. 56%) revealed a slightly higher prevalence via our dataset and dyslipidemia had a lower prevalence (95% vs. 50%). All three demonstrated good discriminatory power. Notably, neurological manifestations, which are highly significant in type A disease, are underrepresented in our dataset, as the prevalence of ASMD subtypes varies, with type B being the most common, followed by A/B, and then type A. Our expert chart review revealed learning disabilities as the most common neurological manifestation, occurring in 40% of cases. Among the skeletal features, growth restriction was the most common feature in our dataset (50% affected). However, growth restriction in childhood is a common sign of several lysosomal storage disorders and other (rare) diseases and often remains undiagnosed for years [30, 31]. Overall, in comparison with the literature, our survey data allowed us to capture a wider range of symptoms and manifestations reported by international patients. These profiles provide valuable insights into the diverse clinical phenotypes of ASMD.

The combination of two or more symptoms strengthens the suspicion of ASMD. This implies that the predictive power of a single factor is insufficient and that at least two relevant symptoms are required for accurate prediction, similar to other SITs for rare diseases [7, 8]. Relying on a single characteristic for screening purposes results in a lower predictive value and more false positives or missed diagnoses. For example, 500,000 individuals need to be screened for dyslipidemia, which occurs in approximately 50% of ASMD patients, given the low prevalence of ASMD cases, with an estimation of 1:250,000 individuals in the general population [32]. Therefore, the use of models that integrate multiple symptoms and clinical parameters, as demonstrated in our SIT, enhances the accuracy and reliability of ASMD diagnosis.

While the SIT was designed with the goal of enabling earlier and more accurate diagnosis of ASMD, it is important to acknowledge that the tool was developed using clinical data collected after symptom onset, with a mean age at data collection of approximately 20 years and a mean age at symptom onset of 8 years in the ASMD cases. This time gap reflects the well-documented diagnostic delay in ASMD and highlights a key challenge in rare disease recognition. However, the features included in the SIT are typically present early in the disease course. Therefore, although the tool was derived from postonset data, the included variables are early clinical signs that could support timely suspicion and referral if recognized and interpreted appropriately in clinical practice. Prospective validation in younger populations and earlier stages of disease will be essential to fully establish the tool’s utility in promoting truly early diagnosis.

The utilization and impact of diagnostic tools in clinical practice are still largely unknown [7, 8, 19–21]. Thus, we devised a novel strategy with our tool defining the target audience, suggesting further diagnostic questions, informing the probability threshold for ASMD evaluation, and enabling patient participation. To the best of our knowledge, this methodology is innovative. Other tools have not defined target audiences or considered other diseases. This method allows effective capture of potential ASMD manifestations in various medical settings and provides a foundation for further diagnosis and treatment.

Proof of the tool’s significant impact on diagnostic procedures has yet to be demonstrated, although evidence supports its potential. The experience of healthcare professionals with the SIT, which is intended to be implemented as a digital decision-support instrument, will be evaluated iteratively and incorporated to assess the reliability of the risk prediction factor. Limitations are related to the retrospective data collection. This could be refined with future data generation. A follow-up study will assess the acceptability of this strategy and the impact of the tool’s availability in the setting of hepatosplenomegaly. If successful, additional application examples will be developed. Importantly, the SIT will be continuously refined as additional clinical data and user experience become available, allowing iterative improvement of its predictive performance and clinical utility.

## Conclusion

We present a novel SIT for the early and accurate diagnosis of ASMD on the basis of data from 908 individuals. The tool demonstrates high discriminating power between ASMD cases, controls, and non-cases. Its adaptation to a specific medical specialty further strengthens the suitability of our tool for use in everyday clinical practice.

## Supplementary Information

Below is the link to the electronic supplementary material.

Supplementary Material 1

## Data Availability

The retrospective questionnaire that supports the findings of this study is available in the supplemental information.

## References

[1] Schuchman EH. The pathogenesis and treatment of acid sphingomyelinase-deficient Niemann-Pick disease. Int J Clin Pharmacol Ther. 2009;47(Suppl 1):S48–57. 10.5414/cpp47048. 20040312 10.5414/cpp47048

[2] Schuchman EH, Wasserstein MP. Types A, B Niemann-Pick disease. Best Pract Res Clin Endocrinol Metab. 2015;29(2):237–47. 10.1016/j.beem.2014.10.002. 25987176 10.1016/j.beem.2014.10.002

[3] McGovern MM, Avetisyan R, Sanson BJ, Lidove O. Disease manifestations and burden of illness in patients with acid sphingomyelinase deficiency (ASMD). Orphanet J Rare Dis. 2017;12(1):41. 10.1186/s13023-017-0572-x. 28228103 10.1186/s13023-017-0572-x PMC5322625

[4] McGovern MM, Dionisi-Vici C, Giugliani R, Hwu P, Lidove O, Lukacs Z, et al. Consensus recommendation for a diagnostic guideline for acid sphingomyelinase deficiency. Genet Med. 2017;19(9):967–74. 10.1038/gim.2017.7. 28406489 10.1038/gim.2017.7 PMC5589980

[5] Keam SJ. Olipudase Alfa: First Approval. Drugs. 2022;82(8):941–7. 10.1007/s40265-022-01727-x. 35639287 10.1007/s40265-022-01727-x

[6] Diaz GA, Jones SA, Scarpa M, Mengel KE, Giugliani R, Guffon N, et al. One-year results of a clinical trial of olipudase alfa enzyme replacement therapy in pediatric patients with acid sphingomyelinase deficiency. Genet Med. 2021;23(8):1543–50. 10.038/s41436-021-01156-3. Epub 2021 Apr 19. 33875845 10.1038/s41436-021-01156-3 PMC8354848

[7] Wijburg FA, Sedel F, Pineda M, Hendriksz CJ, Fahey M, Walterfang M, et al. Development of a suspicion index to aid diagnosis of Niemann-Pick disease type C. Neurology. 2012;78(20):1560–7. 10.212/WNL.0b013e3182563b82. Epub 2012 Apr 18. 22517094 10.1212/WNL.0b013e3182563b82

[8] Pineda M, Mengel E, Jahnová H, Héron B, Imrie J, Lourenço CM, et al. A Suspicion Index to aid screening of early-onset Niemann-Pick disease Type C (NP-C). BMC Pediatr. 2016;16:107. 10.1186/s12887-016-0641-7. 27449637 10.1186/s12887-016-0641-7 PMC4957867

[9] Venables WNR. B D. Modern Applied Statistics with S. 4 ed. New York: Springer; 2002.

[10] Austin PC, Steyerberg EW. Events per variable (EPV) and the relative performance of different strategies for estimating the out-of-sample validity of logistic regression models. Stat Methods Med Res. 2017;26(2):796–808. 25411322 10.1177/0962280214558972 PMC5394463

[11] Peduzzi P, Concato J, Kemper E, Holford TR, Feinstein AR. A simulation study of the number of events per variable in logistic regression analysis. J Clin Epidemiol. 1996;49(12):1373–9. 8970487 10.1016/s0895-4356(96)00236-3

[12] Liaw A, Wiener M. Classification and regression by randomForest. R News. 2001;23.

[13] Wasserstein M, Lachmann R, Hollak C, Arash-Kaps L, Barbato A, Gallagher RC, et al. A randomized, placebo-controlled clinical trial evaluating olipudase alfa enzyme replacement therapy for chronic acid sphingomyelinase deficiency (ASMD) in adults: One-year results. Genet Med. 2022;24(7):1425–36. 35471153 10.1016/j.gim.2022.03.021

[14] Lin S, Nateqi J, Weingartner-Ortner R, Gruarin S, Marling H, Pilgram V, et al. An artificial intelligence-based approach for identifying rare disease patients using retrospective electronic health records applied for Pompe disease. Front Neurol. 2023;14:1108222. 37153672 10.3389/fneur.2023.1108222 PMC10160659

[15] Burlina AB, Polo G, Rubert L, Gueraldi D, Cazzorla C, Duro G, et al. Implementation of Second-Tier Tests in Newborn Screening for Lysosomal Disorders in North Eastern Italy. Int J Neonatal Screen. 2019;5(2):24. 33072983 10.3390/ijns5020024 PMC7510225

[16] Sontag MK, Miller JI, McKasson S, Sheller R, Edelman S, Yusuf C, et al. Newborn screening timeliness quality improvement initiative: Impact of national recommendations and data repository. PLoS ONE. 2020;15(4):e0231050. 32240266 10.1371/journal.pone.0231050 PMC7117765

[17] Arslan N, Coker M, Gokcay GF, Kiykim E, Onenli Mungan HN, Ezgu F. Expert opinion on patient journey, diagnosis and clinical monitoring in acid sphingomyelinase deficiency in Turkey: a pediatric metabolic disease specialist’s perspective. Front Pediatr. 2023;11:1113422. 37435168 10.3389/fped.2023.1113422 PMC10330960

[18] Cox GF, Clarke LA, Giugliani R, McGovern MM. Burden of Illness in Acid Sphingomyelinase Deficiency: A Retrospective Chart Review of 100 Patients. JIMD Rep. 2018;41:119–29. 29995201 10.1007/8904_2018_120 PMC6122055

[19] Yaffe MJ, Wolfson C, Lithwick M, Weiss D. Development and validation of a tool to improve physician identification of elder abuse: the Elder Abuse Suspicion Index (EASI). J Elder Abuse Negl. 2008;20(3):276–300. 18928055 10.1080/08946560801973168

[20] Mignarri A, Gallus GN, Dotti MT, Federico A. A suspicion index for early diagnosis and treatment of cerebrotendinous xanthomatosis. J Inherit Metab Dis. 2014;37(3):421–9. 24442603 10.1007/s10545-013-9674-3

[21] von Arnim U, Röhl FW, Miehlke S, Jechorek D, Reinhold D, Wex T, et al. Clinical symptom tool that raises the index of suspicion for eosinophilic oesophagitis in adults and drives earlier biopsy for definitive diagnosis. Aliment Pharmacol Ther. 2017;45(3):417–26. 27896821 10.1111/apt.13869

[22] Lepakshi VA. Machine learning and deep learning based AI tools for development of diagnostic tools. In: Computational approaches for novel therapeutic and diagnostic designing to mitigate SARS-CoV-2 infection: Copyright © 2022 Elsevier Inc. All rights reserved.; 2022. pp. 399–420.

[23] McGovern MM, Wasserstein MP, Giugliani R, Bembi B, Vanier MT, Mengel E, et al. A prospective, cross-sectional survey study of the natural history of Niemann-Pick disease type B. Pediatrics. 2008;122(2):e341–9. 18625664 10.1542/peds.2007-3016 PMC2692309

[24] Cassiman D, Libbrecht L, Meersseman W, Wilmer A. Case Report of Gastrointestinal Bleeding in an Adult with Chronic Visceral Acid Sphingomyelinase Deficiency. Case Rep Gastrointest Med. 2019;2019:9613457. 31080679 10.1155/2019/9613457 PMC6475549

[25] McGovern MM, Aron A, Brodie SE, Desnick RJ, Wasserstein MP. Natural history of Type A Niemann-Pick disease: possible endpoints for therapeutic trials. Neurology. 2006;66(2):228–32. 16434659 10.1212/01.wnl.0000194208.08904.0c

[26] Lipiński P, Kuchar L, Zakharova EY, Baydakova GV, Ługowska A, Tylki-Szymańska A. Chronic visceral acid sphingomyelinase deficiency (Niemann-Pick disease type B) in 16 Polish patients: long-term follow-up. Orphanet J Rare Dis. 2019;14(1):55. 30795770 10.1186/s13023-019-1029-1 PMC6387484

[27] Wasserstein MP, Desnick RJ, Schuchman EH, Hossain S, Wallenstein S, Lamm C, et al. The natural history of type B Niemann-Pick disease: results from a 10-year longitudinal study. Pediatrics. 2004;114(6):e672–7. 15545621 10.1542/peds.2004-0887

[28] Wasserstein M, Godbold J, McGovern MM. Skeletal manifestations in pediatric and adult patients with Niemann Pick disease type B. J Inherit Metab Dis. 2013;36(1):123–7. 22718274 10.1007/s10545-012-9503-0

[29] Cassiman D, Packman S, Bembi B, Turkia HB, Al-Sayed M, Schiff M, et al. Cause of death in patients with chronic visceral and chronic neurovisceral acid sphingomyelinase deficiency (Niemann-Pick disease type B and B variant): Literature review and report of new cases. Mol Genet Metab. 2016;118(3):206–13. 27198631 10.1016/j.ymgme.2016.05.001

[30] Haymond M, Kappelgaard AM, Czernichow P, Biller BM, Takano K, Kiess W. Early recognition of growth abnormalities permitting early intervention. Acta Paediatr. 2013;102(8):787–96. 23586744 10.1111/apa.12266 PMC3738943

[31] Atallah A, Butin M, Moret S, Claris O, Gaucherand P, Doret-Dion M. Fetal growth restriction: underdiagnosed condition with non-optimal screening. J Matern Fetal Neonatal Med. 2022;35(25):8237–44. 34420493 10.1080/14767058.2021.1967924

[32] Meikle PJ, Hopwood JJ, Clague AE, Carey WF. Prevalence of lysosomal storage disorders. JAMA. 1999;281(3):249–54. 10.1001/jama.281.3.249. 9918480 10.1001/jama.281.3.249

