# Supplementary material for: A suspicion index tool to aid the diagnosis and treatment of ASMD
Source: Orphanet J Rare Dis. 2026 Mar 27;21:178. doi: 10.1186/s13023-026-04328-z (PMC13147735; doi:10.1186/s13023-026-04328-z)
Supplement: Supplementary file 1 — Supplementary Material 1 [file 13023_2026_4328_MOESM1_ESM.pdf]

One questionnaire per patient (ASMD-positive cases or suspected ASMD cases)

Question	Answer
type of disease	◯ infantile neurovisceral ASMD (NPD A)<br>◯ chronic neurovisceral ASMD (NPD A/B)<br>◯ chronic visceral ASMD (NPD B)<br>◯ ___________________________ [other]
gender	◯ female<br>◯ male
presence of individual manifestation	_______ [YY]
current age/ age at death	_______ [YY]
patients first- or second-degree family history	◯ parent with ASMD<br>◯ sibling with ASMD<br>◯ cousin with ASMD
clinical features: central nervous system	◯ loss of skills<br>◯ hypotonia<br>◯ loss of deep tendon reflexes<br>◯ dysphagia<br>◯ peripheral neuropathy<br>◯ extrapyramidal signs<br>◯ psychiatric symptoms<br>◯ learning disabilities<br>◯ ataxia<br>◯ no neurodegeneration<br>___________________________ [other features]
clinical features: ophthalmologic	◯ ocular cherry red spot<br>◯ macular halo<br>◯ no ocular abnormality
clinical features: gastrointestinal	◯ hepatomegaly<br>◯ splenomegaly<br>◯ cholestatic jaundice<br>◯ feeding difficulties<br>◯ vomiting<br>◯ liver fibrosis<br>◯ portal hypertension<br>◯ abnormal liver function<br>◯ diarrhea<br>___________________________ [other features]
clinical features: pulmonary	◯ interstitial lung disease<br>◯ frequent, recurrent respiratory tract infections<br>◯ aspiration pneumonia<br>___________________________ [other features]
clinical features: cardiac	◯ cardiac valve disease<br>◯ early onset coronary artery disease<br>◯ mixed dyslipidemia with low HDL-C<br>___________________________ [other features]
clinical features: musculoskeletal	◯ bone and joint pain<br>◯ reduced bone density(and pathologic fractures)<br>◯ delayed bone maturation<br>◯ growth restriction in childhood<br>___________________________ [other features]
clinical features: hematologic	◯ thrombocytopenia with bleeding tendencies<br>___________________________ [other features]
clinical features: other	◯ poor sleep<br>◯ irritability<br>◯ headache<br>◯ recurrent ear infections<br>___________________________ [other features]
